# New Technologies' Commercialization: The Roles of the Leader's Emotion and Incubation Support

**DOI:** 10.3389/fpsyg.2021.710122

**Published:** 2021-08-19

**Authors:** Biaoan Shan, Yi Pu, Biao Chen, Shan Lu

**Affiliations:** ^1^School of Management, Jilin University, Changchun, China; ^2^School of Business, Zhengzhou University, Zhengzhou, China

**Keywords:** the leader emotion, passion for work, incubation support, technology commercialization, new venture

## Abstract

New technologies, such as brain-computer interfaces technology, advanced artificial intelligence, cloud computing, and virtual reality technology, have a strong influence on our daily activities. The application and commercialization of these technologies are prevailing globally, such as distance education, health monitoring, smart home devices, and robots. However, we still know little about the roles of individual emotion and the external environment on the commercialization of these new technologies. Therefore, we focus on the emotional factor of the leader, which is their passion for work, and discuss its effect on technology commercialization. We also analyzed the moderating role of incubation support in the relationship between the leader's emotion and technology commercialization. The results contribute to the application of emotion in improving the commercialization of new technologies.

## Introduction

In the digital economy era, the application and commercialization of new emerging technologies, such as brain-computer interfaces technology, advanced artificial intelligence, Cloud Computing, and Virtual Reality technology, are strongly influencing our daily life while creating significant amounts of innovation and entrepreneurship opportunities all over the world. Driven by the fast growth of these new technologies, the industries in distance education, health monitoring, smart home, and robots have grown quickly. Many new ventures in these industries have been created. Obviously, with the development of the digital economy and digital technologies, how to commercialize these new technologies has become an important research topic.

Prior studies have focused on various influencing factors of technology commercialization (TC), including external environmental factors (Li et al., 2008; Chen et al., 2011) and organizational factors, such as firm resources, strategic orientation, inter-departmental cooperation, and technological innovation capabilities (Zahra and Nielsen, 2002; Sohn and Moon, 2003; Park and Ryu, 2015; Jo and Park, 2017). Some scholars explored the moderating factors which may strengthen or weaken the efficiency of organizational elements, including formal and informal integration mechanisms, organizational culture, and environmental dynamic (Zahra and Nielsen, 2002; Park and Ryu, 2015). These studies have made important contributions to the understanding of TC. However, existing research ignores the role of leaders in TC, especially when the effect of the leader's emotion on TC is still unknown.

The organizational structure of new ventures is incomplete and informal (Fultz and Hmieleski, 2021). In the process of commercializing technologies, the leaders of new ventures are the core decision makers. However, they always face many challenges, such as a lack of knowledge and experiences, limited resources, and a rapidly changing environment (Chen, 2009; Sullivan and Marvel, 2011). In this situation, leaders need positive emotion to persist with their goals and conquer these challenges in TC (Baum and Locke, 2004; Connelly and Ruark, 2010). In this study, we focus on a salient emotion, a leader's passion for work, which influences leader's behaviors, including communication, learning, and decision making (Baum and Locke, 2004). It is a critical psychological characteristic of leaders (Huy and Zott, 2019; De Cock et al., 2020) and plays an important role in the application of new technologies. Leaders who are passionate about their work would take an active role and systematically participate in the knowledge management process associated with their work (De Clercq et al., 2013), which, in turn, would have a positive effect on the whole company.

In order to address this gap, this study considers the role of a leader's passion for work in commercializing new technologies in new ventures. Klaukien et al. (2013) argue that the role of emotional factors, especially a leader's passion for work in the innovation decision-making activities, needs to be further explored. To reply to this importance, we explore the role of the leaders, as the boundary spanner connecting customers/markets to technologies that significantly promote organizational technology commercialization, on the TC from an individual emotional characteristic perspective (Markman et al., 2008).

In addition, we suggest that the effect of passion for work on the TC is contingent on incubation support. In other words, we consider incubation support as an important moderating variable. TC involves a set of complex behaviors that result from a combination of organizational elements and support environments (Chen, 2009). However, only a few empirical studies explore the interactive role of a leader's emotional characteristics and support environment in TC. Recently, different types of business incubators have been created. These incubators are diverse in function. Incubators provide policy support, funding, necessary entrepreneurial counseling services, and technical support to new ventures (Bøllingtoft, 2012; Bruneel et al., 2012). Hence, incubators can build a support environment to carry out entrepreneurial activities and affect the TC of new ventures (Davidsson et al., 2006). In this study, we consider incubation support as an external support environment factor. We analyze the effect of the interaction between the leader's emotion and incubation support on technology commercialization.

This study mainly tries to answer the question “how do leaders' passion for work influence TC in new ventures?” and explores the moderating role of incubation support. By doing so, this study mainly makes two contributions to existing literature. First, this study recognizes an emotional characteristic of a leader (passion for work) and its impact on TC. The findings further deepen the research of technology innovation and commercialization and expand the research potential of emotion theories in the business field. Second, we consider incubation support as an external environment and examine its moderating impact on the relationship between a leader's passion for work and TC. Finally, researchers have tried to theorize the role of an incubator in the creation and growth of new ventures (Scillitoe and Chakrabarti, 2010; Wu et al., 2020a). In this study, we consider incubation support as an external environment factor and find that context differences in the strength of incubation support could strengthen the effect of the leader's emotion on TC. By doing so, we extend the theoretical research on the interaction between psychological characteristics and external environmental factors on TC.

We organize the remainder of this study as follows: Section Theoretical Background and Hypotheses proposes our hypotheses based on the literature review on the leader's emotion and TC. Section Methodology presents our research method, including the process of data collection, sample characteristics, measurement of variables, and sample validity. Section Results provides regression analyses of the hypotheses and reports the results. Section Discussion discusses the theoretical and managerial implications, the limitations, and the conclusions of this study.

## Theoretical Background and Hypotheses

### Leader's Positive Emotion: The Passion for Work

From a psychological perspective, an individual's behaviors and activities in their everyday life are accompanied by emotional experience (Russell, 2003). Passion, like sadness, happiness, or joy, is an important emotional experience (Cardon et al., 2005; Prayag et al., 2020). Identity centrality and positive feelings are the two basic elements of passion (Cardon et al., 2009). Studies show that individuals can determine their preferences by repeatedly participating in various activities and continuing to engage in related activities (Vallerand, 2008). In this process, an individual's preference gradually resonates with their self-identity and generates positive emotion (Murnieks et al., 2019; Wu et al., 2020b). Therefore, the leader's positive emotion is stable and sustainable which is different from sadness, happiness, and other emotions (Cardon et al., 2009).

A leader's passion for work is an important emotion that affects the organization (De Clercq et al., 2013; Ma et al., 2017). Passion for work represents an individual's feelings about work and reflects the extent to which an individual enjoys working and gains happiness from investing or participating in work-related activities (De Clercq et al., 2013). Passion for work comes from the internalization of an individual's self-identity, and it can be either active or passive (Cardon et al., 2009; Slemp et al., 2021). Active internalization creates a harmonious passion, while passive internalization creates an obsessive passion (Vallerand, 2008).

Regarding the theoretical research on the passion for work, many studies explore the passion for work of employees, such as the effect of passion for work on employee job satisfaction (Houlfort et al., 2013), employee's perception of work (Lavigne et al., 2014), and employee's performance (Burke et al., 2015). A small number of scholars have focused on the passion of entrepreneurs and managers. Baum and Locke (2004) regard passion as a typical individual characteristic and discuss the passion of entrepreneurs in new ventures. With the promotion of passion, entrepreneurs will positively build their resource skills, shape their entrepreneurial cognition, and improve themselves to achieve growth goals. De Clercq et al. (2013) focused on the relationship between potential entrepreneur passion for work and entrepreneurial intention.

Most scholars believe that this type of positive emotion significantly influences individual cognition and behavioral tendencies. However, only a few studies have discussed the impact of passion for work on innovation activities in new ventures. Klaukien et al. (2013) confirmed that a manager's passion for work has a positive effect on their decision to use new product development opportunities. Strese et al. (2018) studied the passion for inventing in CEOs and argued that passion is beneficial for small and medium enterprises to carry out exploratory innovation. As an important psychological characteristic variable, we still know little about how leaders' passion for work impacts innovation in new ventures.

### Technology Commercialization (TC)

Early studies viewed TC as a process to transform new technologies into products or services that meet market needs. The process includes designing, altering, producing, and marketing (Nevens, 1990; Mitchell and Singh, 1996). Originally the vision of implementing ideas, TC refers to the process of turning technology into profit or value (Siegel et al., 1995; Li et al., 2008). In the digital economy era, improving TC is a key means for new ventures to gain a sustained competitive advantage since new technologies, such as brain-computer interfaces technology, advanced artificial Intelligence, cloud computing, and virtual reality technology, constantly emerge and shape lots of opportunities for creating new ventures.

The capability perspective suggests that TC is an essential organizational level capability that enables a company to apply new technologies to develop products or services, increasing the company's revenues, profits, and competitiveness (Kim et al., 2011). Scholars use frequency, speed, novelty, and patents to measure TC (Zahra and Nielsen, 2002). Chen (2009) divides TC into three dimensions: TC speed, market scope, and technology breadth. TC speed refers to the degree of launching new products or services faster than competitors to obtain high profits. Market scope reflects the extent to which products or services are brought to different markets. The broader the scope, the more a firm can amortize the cost of R&D and marketing. Technology breadth refers to how many related technologies are used in a new product. The more technologies, the more likely a product is to satisfy diverse customer demands.

After exploring the concept and the process of TC (Nevens, 1990), more scholars focus on gaining a competitive advantage by promoting TC capability. Scholars in this stream revealed the roles of various factors in improving TC, such as the external environment (Sohn and Moon, 2003), the characteristics of technologies (Chen et al., 2011), the innovative capability and technological capability (Jiang et al., 2017; Jo and Park, 2017), the internal and external resources of the organization (Zahra and Nielsen, 2002), cooperation and knowledge creation (Lin et al., 2015), and the absorptive capacity (Jo and Park, 2017). They mainly focus on the roles of organizational factors (such as resources and capabilities) within a company and the contingent role of environmental factors. But the literature lacks concern about new ventures' commercialization and the effects of leaders' psychological characteristics in this process.

### The Leader Passion for Work and TC in New Ventures

New ventures engage in opportunity development activities under a high resource-constrained environment (Senyard et al., 2014; Cai et al., 2017; Shan and Lu, 2020), where new ventures must cope with high uncertainty when exploring opportunities (Lu et al., 2021a,b). In this context, positive emotion, for example, passion for work, stimulates a leader's desire to succeed. Thus, they will be more enthusiastic and more actively respond to obstacles that may occur in the process of technology commercialization (Baum and Locke, 2004). Perrewé et al. (2014) found that passion for work reflects an ongoing emotion of an individual's desire to work. Therefore, passion for work would promote a leader to complete the work tasks and proactively seek solutions to overcome obstacles in their daily work. High passion for work drives a leader to cope with resource constraints and reduce uncertainty in technology development. This then improves the technology commercialization capability in new ventures.

Passion for work also triggers learning activities and promotes the acquisition of new knowledge (De Clercq and Pereira, 2020; Gong et al., 2020). For example, De Clercq et al. (2013) found that passion for work relates to an individual's cognitive learning process, making a leader actively participate in high-intensity and systematic knowledge processes. It is crucial for the new ventures that lack prior experiences (Shan and Lu, 2020). Driven by high passion for work, a new venture's leader would continuously update their knowledge systems. For example, the leaders would use creative bricolage to learn how to acquire resources to achieve the mission in the high-resources-limited context (Zott and Huy, 2007; Lurtz and Kreutzer, 2014; Murray et al., 2020).

Passion also strengthens a leader's perception of the ability to complete an important task (Cardon et al., 2009), thus improving a leader's motivation in their daily work (Donahue et al., 2012; Feng and Chen, 2020). Passion for work also increases a leader's self-efficacy, which is essential for the leader since high self-efficacy promotes investment in innovation activities and improves technology commercialization. Therefore, passion triggers a leader to choose appropriate strategies and an organizational structure that match innovative activities, promotes the application of innovative technologies, and strengthens the technological breadth of new products (Strese et al., 2018).

Based on the above arguments, we propose the following hypothesis:

**H1**. A leader's passion for work positively influences the capability of technology commercialization in the new venture.

### The Moderating Role of Incubation Support

As an important form of the organization supporting entrepreneurial activities and promoting innovation, incubators provide shared office spaces and facilities to the leaders of the new ventures (Hackett and Dilts, 2004). They create a highly supportive environment for the new ventures (Bergek and Norrman, 2008; Luke et al., 2019), which will positively enhance the probability of new ventures' success (Chen, 2009; Scillitoe and Chakrabarti, 2010; Haugh, 2020). Incubators have various forms. There are incubators established by large companies (Evald and Bager, 2008), science and technology parks based on universities or research institutions (McAdam and McAdam, 2008), and business incubators established by private companies (entrepreneurs) or government agencies (Tello et al., 2012). Incubators can also be divided into diversified incubators and specialized incubators (Schwartz and Hornych, 2010).

In general, the incubation supports new ventures in three ways: providing basic services such as shared spaces and facilities (Grimaldi and Grandi, 2005); providing commercial services such as financial support, management consulting, and business contacts (Scillitoe and Chakrabarti, 2010); and providing technical services such as technical consulting, technology transfer, and intellectual property protection (Bergek and Norrman, 2008; Scillitoe and Chakrabarti, 2010). All the support can greatly reduce the transaction costs of commercializing new technologies. This then increases the survival rate and the success of a technology or a product innovation (Tello et al., 2012).

Incubators can be seen as an innovative system to provide a supportive environment to facilitate the acquisition of information and resources for new ventures (Schwartz and Hornych, 2010). The formal and informal social network inside and outside the incubators offers potential opportunities for new ventures (Nijssen and Michel, 2017). Incubation support becomes an important driving force for the creation and growth of new ventures and actively influences them to conduct innovative activities and exploit business ideas (Davidsson et al., 2006; Schwartz and Hornych, 2010). In this context, new ventures are more likely to succeed in technology commercialization with incubation support.

As mentioned above, a leader's passion for work plays an important role in the process of TC for new ventures. Incubation support provides a more friendly external environment for passionate leaders of new ventures to cope with their challenges (Bergek and Norrman, 2008). Environmental support has significant implications for leaders because passion for work is easily influenced by the external environment (Zigarmi et al., 2009). If one new venture is in an incubator, the leader has more channels to acquire resources, as well as basic services, and other commercial and technical support (Bøllingtoft and Ulhøi, 2005).

The key to technology commercialization is how to match technology and market requirement. A leader of a new venture always lacks market experience (Li and Zhang, 2007). Incubators guide these leaders of the new ventures to develop products or services to meet the needs of the customer. Many specialized incubators have a deep understanding of a specific industry with experience in the technical sectors, which can provide more specialized services in technology development and market operating (Schwartz and Hornych, 2010). These incubators can also provide the necessary technical advisory services (McAdam and McAdam, 2008) that allow the new ventures to meet the requirement of the market (Chen, 2009). Such a support environment greatly reduces the time and cost of commercialization. Therefore, incubation support promotes passionate leaders to focus more on the market-oriented development of technology and improve efficiency.

New technologies' commercialization is a complex process (Chen et al., 2011). The complex and innovative network system generated by an incubator promotes the flow of information and knowledge, which triggers the learning activities at different levels, including interorganizations learning, organizational learning, and employee learning. As Tötterman and Sten (2005) revealed, the incubators positively support new ventures acquiring basic knowledge in terms of technology commercialization processes. The leaders with a higher passion for work may more actively participate in relevant learning activities driven by the technology commercialization with the incubation support.

Therefore, we propose that:

**H2**. Incubation support strengthens the positive relationship between a leader's passion for work and the new venture's technology commercialization capability.

**Figure 1:**
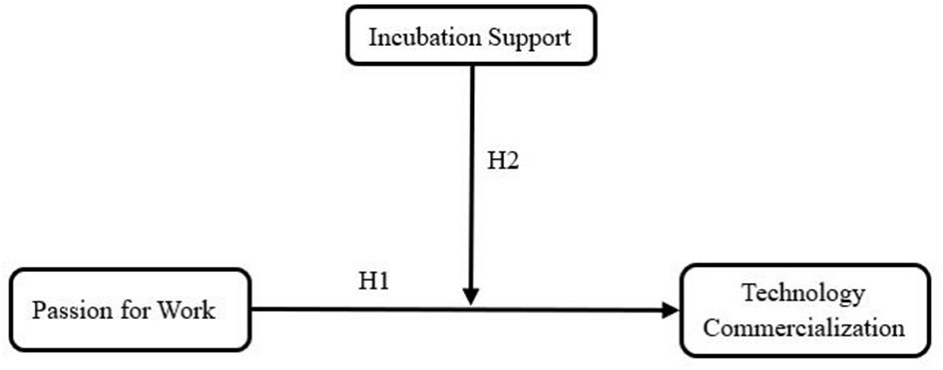
The research model.

Thus, we propose our research model in Figure 1.

## Methodology

### Data Collection and Samples

In order to verify the theoretical hypotheses, we conducted a questionnaire survey in February 2018 in Beijing, Tianjin, Hangzhou, Shanghai, and other cities in the Yangtze River Delta regions, where entrepreneurial activities are relatively active in China. Also, the application of new emerging digital technologies is prevailing there. Driven by the new technologies, many new ventures in sectors such as smart healthcare, smart home devices, and online education are created. Therefore, we considered the new ventures in these regions of China as the sample firms.

Before the formal survey, the authors conducted a pilot test in several companies. According to the feedback of these interviews, we revised and improved the language and items of the core variables to avoid ambiguity in the survey. Subsequently, we randomly distributed questionnaires to companies in these areas mentioned above. We used different ways to collect data. Most samples were collected by email, while the other paper questionnaires were collected during the interview of research team members.

We sent about 500 questionnaires and received 243 questionnaires back. Our research focused on new ventures companies with a registration time within 10 years (Milanov and Fernhaber, 2009). Thus, we deleted the questionnaires from companies who had been registered for more than 10 years. At the same time, we also eliminated the questionnaires with a total missing value more than 25%. In the end, 140 valid samples remained and the characteristics are shown below 0.83 samples (59.28%) are from the Yangtze River Delta region, 57 samples come from other regions such as Beijing and Tianjin which account for 40.72%, 92 samples (65.71%) are from technological sectors such as computer science, new energy, new materials, and software industry, while 48 samples are from non-technological sectors which account for 34.29%. In terms of the number of employees, 84 samples have at least 50 employees and 56 samples (40%) have <50 employees.

### Measures

The measures of the variables in this paper mainly draws on prior mature scales. Likert 7-point was used in the survey (1 is very low or extremely disagree, 7 is very high or extremely agree). In order to ensure the reliability of measurements, we mainly use the scales that are tested by other prior researchers. We translated all the items into Chinese and then translated them back to English independently by different professionals. This may ensure the accuracy of measures.

#### The Capability of Technology Commercialization (TC)

According to Chen (2009), we consider three dimensions: the speed of technology commercialization, market scope, and technology width. We use eight items to measure TC. These include, the capability to quickly develop products, the capability to quickly introduce products/services to the markets, and the capability to improve existing products/services to adapt to different markets.

#### A Leader's Passion for Work (PW)

This variable measurement was developed based on mature studies. We drew on the measurement of Baum and Locke (2004) and De Clercq et al. (2013). Four items were used to reflect passion for work. These included “I like to work hard” or “I am looking forward to returning to work when I am not working.” The scale was used and well tested by prior studies.

#### Incubation Support (IS)

Following the study of Chen (2009), we set a virtual variable to measure incubation support. We asked the leader whether their firms are supported by an incubator, where 0 represents the company has not received any support from an incubator, while 1 means that the company has received support from an incubator.

#### Control Variables

We used five control variables in this study: the number of employees (1–20, 21–50, 51–200, and more than 200), the age of ventures, work experience of a leader, educational background of a leader (high school or below, junior college, bachelor's degree, and master or Ph.D. degree), and the industry. Among these, we set work experience variable based on the working years of a leader in the sector and we set a dummy variable for “industry” based on whether it belongs to the technology industry (0 means non-technology-based industry and 1 means technology-based industry).

We tested the validity of the sample using a confirmatory factor analysis. The results show that the value of factor loading for the variables is more than 0.7. We also calculated the Cronbach's alpha coefficient which exceeds 0.8. The results in Table 1 show good reliability and validity. The Harman's one-factor test was conducted. The result showed that the largest factor only explained 33.76% of the entire variance. According to the view of Podsakoff and Organ (1986), the problem of common method bias is not significant.

**Table 1:** The results of confirmatory factor analysis and Cronbach's alpha coefficients.

**Variables**	**Items**	**Factor loading**	**Cronbach's alpha**
TC	TC1	0.801	0.925
	TC2	0.895	
	TC3	0.841	
	TC4	0.869	
	TC5	0.826	
	TC6	0.815	
	TC7	0.857	
	TC8	0.859	
PW	PW1	0.918	0.870
	PW2	0.914	
	PW3	0.707	
	PW4	0.869	

## Results

The results of the descriptive statistics and the correlations are shown in Table 2. The coefficients of Pearson correlation show a significant correlation between passion for work and technology commercialization. We use multiple linear regression analysis to test the hypotheses. We use the VIFs (variance inflation factors) to test multicollinearity, and the results showed no obvious multicollinearity problem.

**Table 2:** Descriptive statistics and correlation analysis.

**Variable**	**1**	**2**	**3**	**4**	**5**	**6**
Number of employees	1					
Age	0.254^**^	1				
Work experience	0.144	0.183^*^	1			
Education background	0.115	−0.070	−0.210^*^	1		
PW	−0.086	−0.157	−0.203^*^	−0.098	1	
TC	0.117	0.046	0.217^*^	−0.030	0.230^**^	1
Mean	2.93	5.06	7.66	2.93	5.18	4.58
S. D.	0.871	2.861	6.258	0.892	1.071	1.238

* *p < 0.05*,

** *p < 0.01*.

*Dummy variables including industry and incubation support are not shown in the table*.

We built three regression models (see Table 3). Model 1 tests the effects of control variables on TC while model 2 examines the effect of a leaders' passion for work on TC. The results show that the coefficient of passion for work is 0.234 (*p* < 0.05, model 2). Therefore, Hypothesis 1 is supported. To test the moderating effects of incubation support, this study built model 3. The regression analysis shows that the coefficient of passion for work is 0.205 (*p* < 0.05, model 3), and the coefficient of the interaction between passion for work and incubation support is 0.553 (*p* < 0.001, model 3). These results support Hypothesis 2.

**Table 3:** The results of linear regression analysis.

	**Dependent variable: TC**		
	**Model 1**	**Model 2**	**Model 3**
Number of employees	0.055	0.061	0.048
Age	−0.103	−0.071	−0.016
Work experience	0.200	0.243^*^	0.164
Education background	0.080	0.114	0.063
Industry	−0.070	−0.080	−0.017
**Independent variable**			
PW		0.234^*^	0.205^*^
**Interaction**			
IS			−0.071
PW × IS			0.553^***^
R^2^	0.056	0.107	0.397
Adj-R^2^	0.001	0.043	0.339
F Value	1.013	1.678^*^	6.757^***^

* *p < 0.05*,

*** *p < 0.001*.

## Discussion

TC requires a series of complex processes such as intuitive imagination, technology cultivation, design, and product testing (Lin et al., 2015). The findings show that a leader's passion for work is essential in improving TC, which is consistent with the study by Klaukien et al. (2013). To increase the success rate of TC, many scholars paid attention to exploring the effect factors of TC (Zahra and Nielsen, 2002; Sohn and Moon, 2003; Li et al., 2008; Chen et al., 2011; Park and Ryu, 2015; Jo and Park, 2017). However, they ignored the psychological characteristics of founders and the support environment of incubators. TC is a complex process, and many challenges exist in the process. The leader's passion for work is essential in improving TC.

Meanwhile, TC needs different types of resources. For new ventures, they generally lack these resources. Incubators provide a support environment for founders to cope with the challenge, which improves the TC. Hence, this research explored the effects of internal factor (the passion for work) and external factor (incubation support) on TC and found several valuable findings.

A leader with high passion for work promotes knowledge and skills enabling them to overcome potential difficulties (Baum and Locke, 2004; Perrewé et al., 2014). The flexible organizational structure of a new venture means that the leader is always close to other members. A leader's positive emotions can transmit to others through social comparison and emotional imitation (Breugst et al., 2012). A leader who is full of passion for work may encourage other members to work hard and enhance job satisfaction.

We also find that incubators provide a support environment for new ventures, which is a benefit for leaders to promote the capability of TC. Incubator, as a special form of organization, creates a support environment for the TC of new ventures by directly or indirectly providing various services or resources including basic services, commercial support, and technical support (Bøllingtoft and Ulhøi, 2005; Adlesic and Slavec, 2012). This put leaders in a friendly work environment, which stimulates their enthusiasm for participation in technological development and application.

Hence, leaders should flexibly use such incubation supports. For example, they can frequently contact the managers of incubators and utilize shared information and resources. Incubators exist as intermediaries connecting new ventures and resource owners. The leaders also have more opportunities to learn how to meet wider market demands based on the new technologies. It is obvious that the capability to commercialize technology will be strengthened by the support from incubators.

## Implications

The findings of this study have several managerial implications and theoretical implications.

### Theoretical Implications

First, existing research has ignored the role of leader's emotional characteristic in TC (Klaukien et al., 2013). We identify a leader's passion for work as a crucial internal emotional characteristic and examine its effect on TC of new venture, while previous studies have mainly focused on the effects of external environmental factors and organizational factors on technology commercialization (e.g., Sohn and Moon, 2003; Lin et al., 2015; Kirchberger and Pohl, 2016). Based on the characteristics of a new venture, we identify a leader's passion for work as an important impactor and link it to TC. In doing so, we demonstrated that a leader's emotional characteristic plays an important role in the process of innovation, which has important implications for future research on individual emotional factors and innovation of new ventures.

Second, this study casts light on the contextual factor when explaining why some new ventures have more difficulty achieving achieving success than others in technology commercialization. In this regard, previous researchers remarked that the formal/informal integration of resources (Zahra and Nielsen, 2002), and technological turbulence (Li et al., 2008) create the differences. We identify incubation support as a new contextual factor and test its moderating effect on the relationship between a leader's passion for work and TC. Although the scholars have recognized the roles of incubators in the creation and growth of new ventures (e.g., Markman et al., 2008; Schwartz and Hornych, 2010), only a few scholars have used empirical research to analyze the role of interaction of emotional variables and incubation support in TC of new ventures. Our findings suggest that incubators not only influence entrepreneurial activities, but also new ventures' innovation and TC.

### Managerial Implications

First, this research investigates a leader's positive emotion and its effect on TC of a new venture. The finding encourages a new ventures' managers or founders to foster and utilize their passion for work, especially for technology-based new ventures. It may improve the probability of success when the top managers or founders have high passion for work since high uncertainty exists in the process of TC. Therefore, whether an individual has passion for the work could be an evaluation standard to hire a new CEO or manager.

Second, the leaders of new ventures should seek the support of incubators. Different types of incubators, including state-owned incubators/private incubators or diversified incubators/specialized incubators (Schwartz and Hornych, 2010; Tello et al., 2012), provide various resources, knowledge, and services for new organizations located in them. It is an important decision for new ventures to start their business in incubator. In order to improve the speed and success rate of TC, leaders (managers or founders) need to frequently communicate with the managers of incubators to get support from them. For the new ventures that are not located in incubators, their leaders should create relations with some incubators near them. The business network of incubators is also beneficial for leaders of those organizations to facilitate technology commercialization.

Finally, more and more digital technologies are emerging and triggering new opportunities in various industries. Hence, we suggest the governments or their agencies encourage the creation of incubators by providing policy and financial support. Because of the important role of incubators in the application and commercialization of new technologies, more polices should be introduced and more financial funds should be created to develop incubators. Furthermore, government officials should connect with the managers of incubators and the leaders of new ventures directly to understand what is most needed. By doing so, the overall failure rate of TC may be reduced significantly.

## Limitations and Future Research

This study also has limitations. We only explore the direct impact of a leader's passion for work on technology commercialization of the new venture. As a psychological factor, passion for work may affect individual cognition and thus have an indirect impact on organizational innovation. Therefore, future studies may focus on revealing these paths and mechanisms of how a leader's passion for work influences technology commercialization from a cognitive perspective. In addition, other emotional characteristics of leaders, such as happiness or sadness, may also have effects on technology commercialization of a new venture. Therefore, future research should explore the role of these emotional characteristics. Furthermore, considering the more detailed and professional support provided by different types of incubators, such as state-owned/private, specialized/diversified, and university science parks, future researchers can analyze whether different types of incubators could support TC in different ways.

## Conclusion

This study explores the relationship between a leader's positive emotion and technology commercialization of new ventures. Based on a sample of 140 new ventures from China, we find that as a typical emotional characteristic, a leader's passion for work is an important driving force for TC of new ventures. The positive emotion promotes the successful rate of commercializing the emerging technologies such as brain-computer interfaces technology, advanced artificial intelligence, Cloud Computing, and Virtual Reality technology. We also find incubation support provides a friendly external environment for new ventures, and significantly strengthens the role of a leader's passion for work in TC.

## Data Availability Statement

The raw data supporting the conclusions of this article will be made available by the authors, without undue reservation.

## Ethics Statement

The studies involving human participants were reviewed and approved by Department of Technology Economy and Management, Jilin University. The patients/participants provided their written informed consent to participate in this study. Written informed consent was obtained from the individual(s) for the publication of any potentially identifiable images or data included in this article.

## Author Contributions

All authors listed have made a substantial, direct and intellectual contribution to the work, and approved it for publication.

## Conflict of Interest

The authors declare that the research was conducted in the absence of any commercial or financial relationships that could be construed as a potential conflict of interest.

## Publisher's Note

All claims expressed in this article are solely those of the authors and do not necessarily represent those of their affiliated organizations, or those of the publisher, the editors and the reviewers. Any product that may be evaluated in this article, or claim that may be made by its manufacturer, is not guaranteed or endorsed by the publisher.
